# Association between vaccination status and severe health consequences among community-dwelling COVID-19 patients during Omicron BA.1/BA.2 and BA.5-predominant periods in Japan

**DOI:** 10.1265/ehpm.23-00061

**Published:** 2023-06-07

**Authors:** Kimiko Tomioka, Kenji Uno, Masahiro Yamada

**Affiliations:** 1 Nara Prefectural Health Research Center, Nara Medical University, Nara, Japan; 2 Chuwa Public Health Center of Nara Prefectural Government, Nara, Japan; 3 Department of Infectious Diseases, Minami-Nara General Medical Center, Nara, Japan

**Keywords:** COVID-19, SARS-CoV-2, Vaccination, Severe outcomes, Omicron, BA.5, Community-based study, Japan

## Abstract

**Background:**

Many previous studies have reported that COVID-19 vaccine effectiveness decreased over time and declined with newly emerging variants. However, there are few such studies in Japan. Using data from a community-based retrospective study, we aimed to assess the association between vaccination status and severe COVID-19 outcomes caused by the Omicron variant, considering the length of time since the last vaccination dose.

**Methods:**

We included all persons aged ≥12 diagnosed with COVID-19 by a doctor and notified to the Chuwa Public Health Center of Nara Prefectural Government during the Omicron BA.1/BA.2 and BA.5-predominant periods in Japan (January 1 to September 25, 2022). The outcome variable was severe health consequences (SHC) (i.e., COVID-19-related hospitalization or death). The explanatory variable was vaccination status of the individuals (i.e., the number of vaccinations and length of time since last dose). Covariates included gender, age, risk factors for aggravation, and the number of hospital beds per population. Using the generalized estimating equations of the multivariable Poisson regression models, we estimated the cumulative incidence ratio (CIR) and 95% confidence interval (CI) for SHC, with stratified analyses by period (BA.1/BA.2 or BA.5) and age (65 and older or 12–64 years).

**Results:**

Of the 69,827 participants, 2,224 (3.2%) had SHC, 12,154 (17.4%) were unvaccinated, and 29,032 (41.6%) received ≥3 vaccine doses. Regardless of period or age, there was a significant dose-response relationship in which adjusted CIR for SHC decreased with an increased number of vaccinations and a longer time since the last vaccination. On the one hand, in the BA.5 period, those with ≥175 days after the third dose had no significant difference in people aged 65 and older (CIR 0.77; 95% CI, 0.53–1.12), but significantly lower CIR for SHC in people aged 12–64 (CIR 0.47; 95% CI, 0.26–0.84), compared with those with ≥14 days after the second dose.

**Conclusion:**

A higher number of vaccinations were associated with lower risk of SHC against both BA.1/BA.2 and BA.5 sublineages. Our findings suggest that increasing the number of doses of COVID-19 vaccine can prevent severe COVID-19 outcomes, and that a biannual vaccination is recommended for older people.

**Supplementary information:**

The online version contains supplementary material available at https://doi.org/10.1265/ehpm.23-00061.

## Graphical Abstract

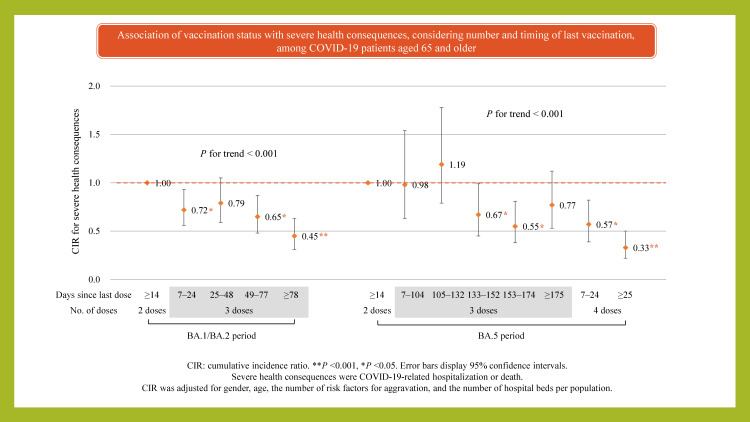

## Background

Since the WHO declared a public health emergency of international concern on January 30, 2020, the Coronavirus disease 2019 (COVID-19) has become a global pandemic, but the spread of COVID-19 in Japan remained at a relatively low level in comparison to other countries [1]. However, since the outbreak of severe acute respiratory syndrome coronavirus 2 (SARS-CoV-2) Omicron sublineage BA.1 in January 2022, the number of infected people in Japan has increased rapidly, becoming the highest in the world in late July 2022 [2]. Because vaccine effectiveness (VE) against COVID-19 in Japan was unknown, using data from COVID-19 patients during the Delta variant and Omicron sublineage (BA.1 and BA.2) predominance, we previously reported that higher vaccination doses were associated with prevention of COVID-19-related health outcomes not only in the Delta variant, but also in Omicron BA.1 and BA.2 sublineages [3]. After that, the Omicron BA.1 and BA.2 sublineages were replaced with the Omicron BA.5 sublineage, which accounts for the majority of COVID-19 cases in Japan since July 2022 [4]. The SARS-CoV-2 continues to change, and even if it is the same Omicron variant, different sublineages may vary in the degree to which they can evade vaccine-induced immunity and in the severity of infection [5, 6]. Notably, previous studies have reported that Omicron BA.5 sublineage is more capable of escaping vaccine-induced immunity than Omicron BA.1 and BA.2 sublineages [6, 7]. Therefore, it is necessary to evaluate the association between vaccination status and severe COVID-19 caused by Omicron BA.5 sublineage among the Japanese population. In addition, in our previous study [3], only the number of vaccinations could be evaluated regarding vaccination status. Future research is needed to assess how long the vaccine will continue to prevent severe disease from COVID19.

In this study, using the same target area and the same data source as our previous study [3], we investigated the association between vaccination status and severe health outcomes during the period from January to September 2022 when the Omicron BA.1/BA.2 and BA.5 sublineages were predominant in Japan, considering not only the number of vaccinations, but also the interval between last vaccination dose and COVID-19 onset.

## Methods

### Data and study participants

**Fig. 1:**
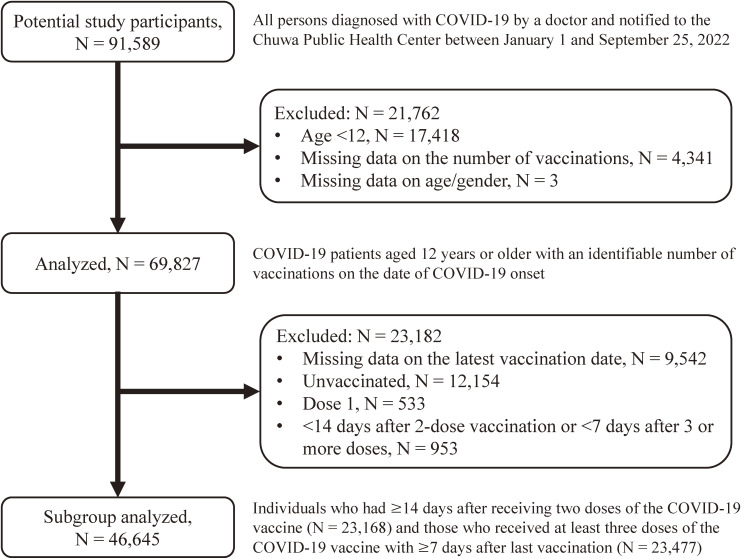
Flow chart of study participants.

We analyzed data from the Health Center Real-time Information-sharing System on COVID-19 (HER-SYS) [8]. The details of the HER-SYS are explained elsewhere [3]. Briefly, HER-SYS is an online system to capture and manage information on COVID-19 patients, such as past medical history and progress of health outcomes. The target area for this study was the jurisdiction of the Chuwa Public Health Center of the Nara Prefectural Government in Japan, which includes 18 municipalities (7 cities, 8 towns, and 3 villages) in the northern part of Nara Prefecture. Potential research subjects were all patients (n = 91,589) who were notified by a doctor to the Chuwa Public Health Center as COVID-19, in accordance with the Infectious Diseases Control Law, between January 1, 2022 and September 25, 2022. The details of the study population are explained elsewhere [3]. In this study, 69,827 persons were analyzed, excluding those under the age of 12, those whose number of vaccinations was unknown, and those whose age/gender was unknown (Fig. 1). That is, persons with missing values were excluded from the analysis. We compared the basic attributes of those who had data on vaccination status and those who had missing data on vaccination status (Additional file 1). Compared to people without missing data, people with missing data on the number of vaccinations were more likely to have severe health consequences, and people with missing data on the last vaccination date were more likely to be younger age, male gender, the BA.5-predominant period, and residents of municipalities with a higher number of hospital beds per population.

### Period of spread of each SARS-CoV-2 Omicron sublineage

Based on the COVID-19 genome analyses published by the National Institute of Infectious Diseases [4], January to June 2022, when Omicron sublineages BA.1 and BA.2 were predominant, was defined as the BA.1/BA.2 period, and July to September 2022, when Omicron sublineage BA.5 accounted for more than 80% of COVID-19 positives, was defined as the BA.5 period. In Japan, the system of reporting all cases of COVID-19 was reviewed, and from September 26, 2022, mandatory notification was limited to those at high risk of severe illness, such as older people [9]. Therefore, because it is difficult to capture all cases of COVID-19 patients after September 26, the BA.5-dominated period was set until September 25, 2022.

### Explanatory variable

The explanatory variable was vaccination status. Based on data available from 30,357 of the study participants on the type of COVID-19 vaccine, BNT162b2 from Pfizer-BioNTech accounted for 71.7%, mRNA-1273 from Moderna for 28.1%, and others for 0.2%. In Japan, bivalent vaccine against the Omicron variant started after September 20, 2022 [10], but in the target area of this research, the Omicron variant-specific vaccine was scheduled to start in October 2022 [11]. Therefore, the COVID-19 vaccine for participants in this study can be assumed to be the original monovalent mRNA-based vaccine.

First, we assessed the number of vaccinations against COVID-19 on the date of COVID-19 onset. The number of days from the last vaccination to onset of COVID-19 was then assessed for a total of 46,645 residents with an identifiable date of last vaccination (i.e., 23,168 individuals who had 14 or more days after receiving two doses of the COVID-19 vaccine and 23,477 individuals who received at least three doses of the COVID-19 vaccine with at least 7 days after the last vaccination) (Fig. 1). The reason for limiting the participants to those who passed 14 days or more after 2-dose vaccination or those who passed 7 days or more after ≥3 doses was that, in previous studies, the period until the acquisition of effective immunity after vaccination with the mRNA-based vaccine was 14 days after the second dose and 7 days after the booster doses [12–14].

Previous studies examining the durability of mRNA-based vaccines against Omicron sublineages divided the time since the last vaccine dose into 4-week [15], 2-month [5], 2/3-month [16], and 120-day [7] intervals, or irregular pattern [17, 18]. Following previous studies, we also considered a method of classifying participants by a certain number of days, but this method was not able to prevent unstable values in groups with a small number because these groups were of unequal size. Therefore, to examine whether the risk of severe health consequences would tend to increase or decrease with increasing interval, we divided the interval from the last vaccination to the onset of COVID-19 equally by the number of participants in each period: the 3-dose recipients were grouped into quartiles during the BA.1/BA.2 period and into quintiles during the BA.5 period. The 4-dose recipients had only the BA.5 period and were divided equally by the median.

### Outcome

The outcome was severe health consequences, i.e., COVID-19-related hospitalization or death. Hospitalized cases were those who were hospitalized between the time they were diagnosed with COVID-19 and the end of the isolation period. Deceased cases were those who were positive for COVID-19 and died during the isolation period. Cases for which HER-SYS data were available indicating that the cause of death was not COVID-19 were excluded from deaths in this study.

### Covariates

Referring to previous studies [19–22], the following variables were identified as potential confounding factors in the association between vaccination status and severe health consequences: gender, age, the number of risk factors for aggravation, and the number of hospital beds per population in the municipality in which the patient resided (hereafter, the number of hospital beds per population). For age, study participants were classified into 4 age groups, 12–59 years, 60–69 years, 70–79 years, and 80 years or older, based on the age at the onset date. Regarding risk factors for aggravation, the following factors were adopted with reference to the COVID-19 medical treatment guidelines [23] and previous studies [20, 22]: chronic respiratory disease, diabetes, chronic kidney disease, hypertension, dyslipidemia, cardiovascular disease, cerebrovascular disease, malignant tumors, smoking, obesity (body mass index ≥30), and pregnancy. The number of risk factors that corresponded to these 11 factors was calculated and classified into three groups: none, one, and two or more. For the number of hospital beds per population, previous studies used the number of hospital beds per population as a healthcare capacity associated with regional variation in the COVID-19 case-fatality ratio [24, 25]. Participants were classified into quartile groups based on the number of hospital beds per population. The number of hospital beds per population was taken from the 2020 Survey of Medical Institutions [26]. Moreover, there was no variable with a variance inflation factor value >2.0, confirming no multicollinearity issues.

### Statistical analysis

Using the generalized estimating equations of the multivariable Poisson regression models after simultaneously adjusting for all covariates, we estimated the adjusted cumulative incidence ratio (CIR) with 95% confidence interval (CI) for severe health consequences. First, we investigated the association between the number of vaccinations and severe health consequences by Omicron sublineage period, with the unvaccinated group as the reference. Next, we assessed the risk of developing severe health consequences in 3- and 4-dose vaccines relative to the 2-dose vaccine by Omicron sublineage period, taking into account the time from the last vaccination to the onset of COVID-19. Moreover, because age is an important confounding factor in the association between vaccination status and severe health outcomes, these analyses were performed separately by age group (i.e., aged 65 and older and aged 12–64 years).

Statistical analyses were performed using the IBM SPSS Statistics Ver. 27 for Windows (Armonk, New York, US), and a significant level was set at 0.05 (two-tailed test).

## Results

Of the 69,827 participants aged 12 and older, the average observation period was 10.1 days (standard deviation, 2.3), the average age was 42.3 (standard deviation, 19.9), the prevalence of older people aged 65 and older was 14.9%, and the male prevalence was 47.2%. For vaccination status, 12,154 participants (17.4%) were unvaccinated, 533 (0.8%) received one vaccine dose, 28,108 (40.3%) received two vaccine doses, 25,582 (36.6%) received three vaccine doses, and 3,450 (4.9%) received four vaccine doses. For the cumulative incidence during the survey period, 2,224 individuals (3.2%) had severe health consequences. Cumulative incidence of people with severe health consequences was 1,139 out of 25,264 (4.5%) in the BA.1/BA.2 period and 1,085 out of 44,563 (2.4%) in the BA.5-predominant period, showing a significant difference (chi-squared test, *P* < 0.001).

Regarding characteristics of the study participants by period (Table 1), young people were more common in the BA.1/BA.2 period. In the BA.5 period, there were more people with aggravation risk factors. There was no difference in gender or the number of hospital beds per population between the BA.1/BA.2 and BA.5 periods.

**Table 1:** Characteristics of the study participants by period

	**Entire** **period**	**Omicron sublineage period**		***P*-value**

		**BA.1/BA.2**	**BA.5**	
	**n = 69,827**	**n = 25,264**	**n = 44,563**	
Gender: male (%)	47.2%	47.2%	47.2%	0.969^a^
Age				
Aged 65 and older (%)	14.9%	13.6%	15.6%	<0.001^a^
Average (SD)	42.3 (19.9)	40.9 (19.7)	43.2 (20.0)	<0.001^b^
Number of aggravation risk factors				<0.001^a^
None	71.8%	73.8%	70.7%	
One	19.1%	19.9%	18.7%	
Two or more	9.0%	6.2%	10.6%	
No. of hospital beds per 10,000 population				0.523^a^
1st quartile group (<80)	23.9%	24.2%	23.7%	
2nd quartile group (80–90)	29.3%	29.1%	29.4%	
3rd quartile group (95–150)	16.9%	16.9%	16.9%	
4th quartile group (>150)	29.9%	29.9%	30.0%	

SD: standard deviation.

^a^*P*-value based on the chi-squared test. ^b^*P*-value based on the t test.

**Fig. 2:**
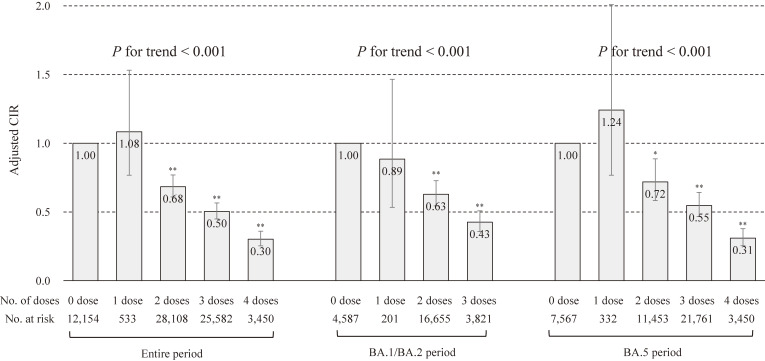
Association of the number of vaccinations with severe health consequences by Omicron sublineage type. CIR: cumulative incidence ratio. **P < 0.001, *P < 0.05. Error bars display 95% confidence intervals. Severe health consequences were COVID-19-related hospitalization or death. CIR in entire period was adjusted for gender, age, the number of risk factors for aggravation, the number of hospital beds per population, and period. CIR by period was adjusted for gender, age, the number of risk factors for aggravation, and the number of hospital beds per population. Vaccination status was based on the number of vaccinations on the day of COVID-19 onset.

Regarding the association between the number of vaccinations and severe health consequences (Fig. 2), during the entire period of the Omicron variant pandemic, the adjusted CIR (95% CI) for severe health consequences was 1.08 (0.77–1.53) in one vaccine dose, 0.68 (0.61–0.77) in two vaccine doses, 0.50 (0.45–0.57) in three vaccine doses, and 0.30 (0.25–0.36) in four vaccine doses, compared to the unvaccinated. Two or more doses were associated with a reduced risk of severe health consequences, and there was a dose-response relationship in which the CIR of severe health consequences decreased with an increasing number of vaccinations (*P* for trend <0.001). After stratified analyses by period (Fig. 2), regardless of the period of the Omicron sublineage, similar results were observed (*P* for trend <0.001 in both periods). After stratified analyses by age and period (Additional file 2), both a significant association and a significant dose-response relationship were observed not only at any period, but also at any age.

Regarding the association of the number and timing of 3- and 4-dose vaccines relative to the 2-dose vaccine with severe health consequences (Table 2), a dose-response relationship was significant in both BA.1/BA.2 and BA.5 periods, in which the risk of severe health consequences decreased with more doses and a longer time since the last vaccination (*P* for trend <0.001 in both periods). On the one hand, those with 25 to 48 days after the third dose in the BA.1/BA.2 period (adjusted CIR = 0.81, 95% CI = 0.62–1.06), those with 7 to 104 days after the third dose in the BA.5 period (adjusted CIR = 0.95, 95% CI = 0.70–1.30), those with 105 to 132 days after the third dose in the BA.5 period (adjusted CIR = 1.11, 95% CI = 0.83–1.48), and those with 175 days or more after the third dose in the BA.5 period (adjusted CIR = 0.76, 95% CI = 0.57–1.01) had no significant difference with those with 14 days or more after the second dose (i.e., the non-booster group). After stratified analyses by age and period, a significant dose-response relationship was observed, regardless of age or period (*P* for trend <0.001 in both periods of aged 65 and older, *P* for trend = 0.004 in the BA.1/BA.2 period of aged 12–64 years, and *P* for trend = 0.007 in the BA.5 period of aged 12–64 years). However, in the BA.5 period, those with 175 days or more after the third dose had no significant difference in the aged 65 and older group (adjusted CIR = 0.77, 95% CI = 0.53–1.12), but a significantly reduced risk of severe health consequences in the aged 12–64 group (adjusted CIR = 0.47, 95% CI = 0.26–0.84), compared with the non-booster group.

**Table 2:** Association of vaccination status with severe health consequences, considering number and timing of last vaccination

**Period**	**No. of** **doses**	**Days since** **last dose**	**All**			**Aged 65 and older**			**Aged 12–64 years**		
		
			**n**	**CIR^a^ (95% CI)**	***P***	**n**	**CIR^a^ (95% CI)**	***P***	**n**	**CIR^a^ (95% CI)**	***P***
**BA.1/BA.2 period**											
	2 doses	≥14	15,303	1.00		1,788	1.00		13,515	1.00	
	3 doses^b^	7–24	810	0.72 (0.56–0.91)	0.006	293	0.72 (0.56–0.93)	0.010	517	0.67 (0.31–1.43)	0.298
		25–48	853	0.81 (0.62–1.06)	0.123	217	0.79 (0.59–1.05)	0.105	636	0.81 (0.43–1.53)	0.517
		49–77	803	0.63 (0.48–0.84)	0.001	194	0.65 (0.48–0.87)	0.004	609	0.55 (0.26–1.17)	0.123
		≥78	833	0.43 (0.31–0.60)	<0.001	266	0.45 (0.31–0.63)	<0.001	567	0.31 (0.12–0.85)	0.022
				*P* for trend <0.001			*P* for trend <0.001			*P* for trend = 0.004	
**BA.5 period**											
	2 doses	≥14	7,865	1.00		216	1.00		7,649	1.00	
	3 doses^c^	7–104	3,444	0.95 (0.70–1.30)	0.757	212	0.98 (0.63–1.54)	0.939	3,232	0.90 (0.57–1.40)	0.630
		105–132	3,445	1.11 (0.83–1.48)	0.480	383	1.19 (0.79–1.78)	0.409	3,062	0.97 (0.63–1.49)	0.871
		133–152	3,497	0.72 (0.53–0.97)	0.029	858	0.67 (0.45–0.99)	0.044	2,639	0.79 (0.48–1.28)	0.334
		153–174	3,449	0.61 (0.46–0.82)	0.001	1,141	0.55 (0.38–0.81)	0.002	2,308	0.68 (0.40–1.16)	0.151
		≥175	3,491	0.76 (0.57–1.01)	0.056	679	0.77 (0.53–1.12)	0.175	2,812	0.47 (0.26–0.84)	0.010
	4 doses^d^	7–24	1,407	0.63 (0.46–0.85)	0.003	1,005	0.57 (0.39–0.82)	0.003	402	0.97 (0.43–2.22)	0.948
		≥25	1,445	0.34 (0.24–0.48)	<0.001	1,176	0.33 (0.22–0.50)	<0.001	269	0.18 (0.03–1.33)	0.093
				*P* for trend <0.001			*P* for trend <0.001			*P* for trend = 0.007	

CI: confidence interval; CIR: cumulative incidence ratio. Analyzed persons are those who have received 2 vaccinations with 14 days or more since the last vaccination and those who have received 3 or more vaccinations with 7 days or more since the last vaccination.

^a^Adjusted for gender, age, the number of risk factors for aggravation, and the number of hospital beds per population. ^b^Quartile groups according to the number of days elapsed from the vaccination date among those who received 3 doses. ^c^Quintile groups according to the number of days elapsed from the vaccination date among those who received 3 doses. ^d^Two groups at the median number of days elapsed from the vaccination date in 4-dose recipients.

## Discussion

Among COVID-19 patients aged 12 years and older during BA.1/BA.2 and BA.5 predominant periods of SARS-CoV-2 Omicron variants in Japan, people who received two or more doses of the COVID-19 vaccine were at lower risk of severe health consequences than those who were not vaccinated, and a dose-response relationship between an increasing number of vaccinations and lower risk of severe health consequences was observed, regardless of the sublineage of the Omicron variant or age group. An analysis based on the time since the last dose showed a trend toward a lower risk of severe health consequences with a longer time since the last dose.

Omicron sublineage VE was previously studied in multiple countries. Some studies have reported that VE against hospitalization wanes about 3–4 months after the third vaccine dose [5, 7], and that VE against BA.5 is lower and wanes more rapidly than earlier Omicron sublineages [6, 7]. Two recent studies demonstrated that the VE of 3 doses against hospitalization or death due to Omicron infections remained durable over time. First, in a study of community residents aged 18 years and older in Hong Kong [27], the effectiveness of the BNT162b2 vaccine was 84% within 3 months after 3 doses and 85% at 4 to 6 months after 3 doses. Second, a test-negative, case-control study in Qatar [28] found that the effectiveness of mRNA COVID-19 vaccines was greater than 90% in ≥7 weeks after 3 doses. Our results are consistent with the results of studies in Hong Kong and Qatar.

Previous studies [6, 28, 29] have pointed out that VE against infection wanes several months after vaccination, while mRNA vaccines provide durable protection against severe outcomes. The reason why the infection-preventing effect weakens after a certain period of time is that mucosal immunity plays a prominent role in defense against respiratory infections, and the nasal IgA responses return to pre-COVID-19 levels after 9 months and are minimally boosted by vaccination [30]. In contrast, systemic immunity plays an important role in preventing progression to severe COVID-19, and plasma IgA and IgG responses remain elevated for at least 12 months and are enhanced by vaccination [30]. This study evaluated the risk of severe health consequences in COVID-19 patients, not the risk of infection. The increased plasma antibody responses after the third dose are maintained over time, which may have reduced the risk of severe health consequence from Omicron infection. In addition, this study used the original monovalent vaccines rather than a bivalent vaccine for Omicron variants. A previous study evaluating the neutralizing activity of bivalent vaccines against each sublineage of the Omicron variants reported that the bivalent vaccine induced higher neutralizing activity against the Omicron BA.5 than the monovalent vaccine [31]. Therefore, because it takes time for the neutralization activity against Omicron BA.5 induced by the monovalent vaccine to reach a high level, the risk of severe health consequences within 132 days after three doses may not have decreased. Future studies are needed to assess the risk of severe outcomes based on bivalent vaccination in Japan.

We discuss below why there was no significant difference between the 3rd and 2nd vaccinations in the Omicron BA.5 period. First, there was only a small number of people aged 65 and older with 132 days or less and 175 days or more after 3 doses. This suggests that insufficient sample power may have led to no significant difference. Second, there was no significant difference in the risk of severe health consequences in older people with 175 days or more after the third dose compared with those who received 2 doses, while younger people had a significant difference between 175 days or more after the third dose and the second dose. Previous studies found that older age was a disadvantage in maintaining humoral immunity [27, 32], suggesting the possibility that the protection against severe COVID-19 is more likely to wane as time passes after the last vaccination in older people than in younger people. Our results support a booster vaccination every six months for older people.

This study has several strengths. First, we assessed the risk of serious health consequences for all infected individuals in the target community. Second, we showed that the COVID-19 vaccine was effective, regardless of the sublineage of the Omicron variant or age. Third, we were able to consider important risk factors for severe COVID-19, including age, comorbidities, smoking, and obesity.

Our study has several limitations. First, although this study was based on patient information reported by the physician who diagnosed COVID-19, if the physician did not administer the vaccine to the patient, the vaccination history was based on information obtained by questioning the patient (or the patient’s family). Therefore, vaccination status may be affected by recall bias, leading to the possibility of misclassification. Second, those with missing data on the number of vaccinations comprised significantly more people with severe health consequences than those whose vaccination status could be identified (Additional file 1). If people who were both unvaccinated and had severe health consequences are selectively excluded from our analyses, our findings may underestimate the association between vaccination status and severe health consequences. Third, although those with a history of COVID-19 acquire immunity to COVID-19 [33, 34], data on the presence or absence of previous infection were lacking in this study. Therefore, our results cannot consider previous infection. If many of the unvaccinated individuals had previous COVID-19 infection, our observed association may have been underestimated. Fourth, hospitalization may be indicated for immunosuppressed post-organ transplant patients and cancer patients undergoing treatment, regardless of the severity of COVID-19 [35]. People unable to manage at home by themselves, such as persons requiring ongoing home care and hemodialysis patients, may also be hospitalized, regardless of the severity of COVID-19 [36]. It should be noted that this study included such hospitalizations not related to the severity of COVID-19. Fifth, unadjusted confounders should be considered. For example, previous studies have reported that the health literacy of the individual is related to COVID-19 vaccine acceptance [37], and that socioeconomic factors (e.g., educational attainment and income) are associated with vaccine hesitancy [38]. However, due to the lack of individual data among the existing data, we were unable to include health literacy and socioeconomic factors as covariates. It is unclear whether the observed associations are overestimated or underestimated by the effects of unadjusted confounders. Sixth, this study has many missing values. In order to avoid under-analysis due to missing values, the multiple imputation method could be used. However, in this study, as shown in Additional file 1, because many of those with an unknown number of vaccinations developed severe health consequences, it was difficult to accept the “missing at random” assumption, which is the premise of the multiple imputation method [39]. Therefore, multiple imputations were not performed in this study, and those with missing values were excluded from the analysis. Given that only those participants without missing values were included in the final analyzed dataset, the association between vaccination status and severe health consequences may have been underestimated. Seventh, during the spread of the Omicron variant, there were cases where patients who were originally eligible for hospitalization were recovering at home because of a shortage of hospital beds [36, 40]. In addition, due to rules on the intervals between vaccinations [10], some people were unable to receive a booster vaccination when the Omicron variant spread. That is, indications for hospitalization may have varied depending on social conditions and the time of year. Therefore, we need to acknowledge that factors other than vaccine effectiveness may have influenced the reduction in the risk of severe disease. Eighth, because genome analyses of COVID-19 patients were not performed in this study, we were unable to accurately estimate the association between vaccination and prevention of severe COVID-19 for each SARS-CoV-2 Omicron sublineage. Ninth, because the target area was limited to 18 municipalities in Nara Prefecture in Japan, caution is required in generalizing to other areas. Finally, the effectiveness of vaccines needs to be judged comprehensively by considering the advantages and disadvantages [41]. However, because the HER-SYS, the data source in this study, did not include information on side effects, we were unable to assess the disadvantages of COVID-19 vaccines. In future research, it is necessary to evaluate the effectiveness of COVID-19 vaccines comprehensively based on both the merits such as the effect of preventing severe disease and the demerits such as side effects.

Despite the above limitations, this study is valuable as the first to demonstrate the protective effect of monovalent COVID-19 vaccines against severe health consequences caused by Omicron sublineage BA.5 infection in Japan. Our findings suggest that increasing the number of doses of COVID-19 vaccine can help prevent severe health consequences in infected people aged 12 years and older. Policy makers should continue to push national programs to increase the number of vaccinations to prevent deaths and hospitalizations from COVID-19. Physicians should encourage patients, especially people at higher risk of developing severe illness, such as older adults, to get additional vaccinations to reduce their risk of severe health consequences from COVID-19.

In conclusion, we found a dose-response relationship between a higher number of vaccinations and lower risk of COVID-19-related hospitalization or death not only during the Omicron BA.1/BA.2-predominant period, but also during the Omicron BA.5-predominant period. Although vaccines have adverse effects as well as positive, our results indicate that increasing the number of vaccinations can reduce the severity of COVID-19, and that a biannual vaccination is recommended for older people.

## Abbreviations

CI: confidence interval
CIR: cumulative incidence ratio
COVID-19: Coronavirus disease 2019
HER-SYS: Health Center Real-time Information-sharing System on COVID-19
SARS-CoV-2: severe acute respiratory syndrome coronavirus
VE: vaccine effectiveness

## Supplementary information

**Additional file 1.** Basic attributes of those whose vaccination status could be identified and those whose vaccination status could not be identified. **Additional file 2.** Association of the number of vaccinations with severe health consequences by age and period.

## Declarations

### Availability of data and materials

The data analyzed in this study are not publicly available.
