# Supplementary material for: Association between vaccination status and severe health consequences among community-dwelling COVID-19 patients during Omicron BA.1/BA.2 and BA.5-predominant periods in Japan
Source: Environ Health Prev Med. 2023 Jun 7;28:35. doi: 10.1265/ehpm.23-00061 (PMC10287986; doi:10.1265/ehpm.23-00061)
Supplement: Supplementary file 1 — Additional file 1. Basic attributes of those whose vaccination status could be identified and those whose vaccination status could not be identified. Additional file 2. Association of the number of vaccinations with severe health consequences by age and period. [file ehpm-28-035-s001.docx]

**Additional file 1.** Basic attributes of those whose vaccination status could be identified and those whose vaccination status could not be identified.

|  | All^a^ (N = 74,168) | |  | People without  missing data on  vaccination status  (N = 60,285) | |  | People with  missing data on  no. of vaccinations  (N = 4,341) | |  | People with  missing data on the  last vaccination date  (N = 9,542) | | *P*-value^b^ |
| --- | --- | --- | --- | --- | --- | --- | --- | --- | --- | --- | --- | --- |
|  | N | % |  | N | % |  | N | % |  | N | % |  |
| People with severe health consequences | 2,583 | 3.5% |  | 1,961 | 3.3% |  | 359 | 8.3% |  | 263 | 2.8% | <0.001 |
| Age: aged 65 and older | 11,027 | 14.9% |  | 9,557 | 15.9% |  | 641 | 14.8% |  | 829 | 8.7% | <0.001 |
| Gender: male | 35,081 | 47.3% |  | 28,147 | 46.7% |  | 2,099 | 48.4% |  | 4,835 | 50.7% | <0.001 |
| Period: Omicron BA.5-predominant period | 47,330 | 63.8% |  | 36,511 | 60.6% |  | 2,767 | 63.7% |  | 8,052 | 84.4% | <0.001 |
| No. of hospital beds per population: 4th quartile group^c^ | 22,994 | 30.1% |  | 17,645 | 29.3% |  | 2,104 | 32.2% |  | 3,245 | 34.0% | <0.001 |

^a^ Limited to people aged 12 and older.

^b^ *P*-value based on the chi-squared test.

^c^ >150 per 10,000 population.

**Additional file 2.** Association of the number of vaccinations with severe health consequences by age and period

| Period | No. of  vaccinations^a^ | Aged 65 and older | | |  | Aged 12–64 years | | |
| --- | --- | --- | --- | --- | --- | --- | --- | --- |
|  |  | n | CIR^b^ (95% CI) | *P* |  | n | CIR^b^ (95% CI) | *P* |
| BA.1/BA.2 period | |  |  |  |  |  |  |  |
|  | Unvaccinated | 227 | 1.00 |  |  | 4,360 | 1.00 |  |
|  | 1 dose | 22 | 0.95 (0.53-1.72) | 0.869 |  | 176 | 0.86 (0.33-2.22) | 0.753 |
|  | 2 doses | 1,999 | 0.66 (0.55-0.79) | <0.001 |  | 14,656 | 0.58 (0.46-0.74) | <0.001 |
|  | 3 doses | 1,176 | 0.46 (0.37-0.56) | <0.001 |  | 2,645 | 0.34 (0.22-0.51) | <0.001 |
|  |  |  | *P* for trend <0.001 | |  |  | *P* for trend <0.001 | |
| BA.5 period | |  |  |  |  |  |  |  |
|  | Unvaccinated | 411 | 1.00 |  |  | 7,156 | 1.00 |  |
|  | 1 dose | 25 | 1.09 (0.64-1.86) | 0.745 |  | 307 | 1.36 (0.51-3.64) | 0.542 |
|  | 2 doses | 292 | 0.70 (0.52-0.94) | 0.017 |  | 11,161 | 0.82 (0.60-1.13) | 0.233 |
|  | 3 doses | 3,680 | 0.51 (0.43-0.61) | <0.001 |  | 18,081 | 0.61 (0.44-0.83) | 0.002 |
|  | 4 doses | 2,554 | 0.29 (0.23-0.35) | <0.001 |  | 896 | 0.46 (0.22-0.94) | 0.033 |
|  |  |  | *P* for trend <0.001 | |  |  | *P* for trend <0.001 | |

CI: confidence interval; CIR: cumulative incidence ratio.

Severe health consequences were COVID-19-related hospitalization or death.

^a^Based on the number of vaccinations on the day of COVID-19 onset.

^b^Adjusted for gender, age, the number of risk factors for aggravation, and the number of hospital beds per population.
